# Efficacy of Gastric Balloon Dilatation and/or Retrievable Stent Insertion for Pyloric Spasms after Pylorus-Preserving Gastrectomy: Retrospective Analysis

**DOI:** 10.1371/journal.pone.0144470

**Published:** 2015-12-10

**Authors:** Jae Seok Bae, Se Hyung Kim, Cheong-il Shin, Ijin Joo, Jeong Hee Yoon, Hyuk-Joon Lee, Han-Kwang Yang, Jee Hyun Baek, Tae Han Kim, Joon Koo Han, Byung Ihn Choi

**Affiliations:** 1 Department of Radiology, Seoul National University Hospital, Seoul, Korea; 2 Institute of Radiation Medicine, Seoul National University Hospital, Seoul, Korea; 3 Department of Surgery, Seoul National University Hospital, Seoul, Korea; 4 Department of Radiology, New Korea Hospital, Gimpo, Gyeonggi-do, Korea; Texas A&M Health Science Center, UNITED STATES

## Abstract

**Purpose:**

We retrospectively investigated the feasibility and clinical efficacy of balloon dilatation and subsequent retrievable stent insertion, when necessitated, for pyloric spasms after pylorus-preserving gastrectomy (PPG).

**Materials and Methods:**

Forty-five patients experiencing pyloric spasms after PPG underwent fluoroscopic balloon dilations to alleviate obstructive symptoms due to delayed gastric emptying. Patients showing poor response to balloon dilation underwent subsequent retrievable stent insertion. Safety of the procedures was analyzed, and subjective symptoms and objective signs of pyloric spasms were analyzed and compared before and after treatment.

**Results:**

Thirty-three patients (73.3%, 33/45) showed good response to balloon dilatation requiring no further treatment (balloon group). Conversely, 12 patients (26.7%, 12/45) showed poor or no response after balloon dilation requiring subsequent stent insertion (stent group). Balloon dilations and/or stent insertions were safely performed in all patients except one patient who suffered a transmural tear after balloon dilatation. In both groups, mean subjective symptom score was significantly improved and mean pyloric canal-to-height of the adjacent vertebral body ratio was significantly increased after the procedures (P <.05).

**Conclusion:**

Balloon dilation is a safe and effective treatment for patients with pyloric spasms after PPG. In patients refractory to balloon dilations, retrievable stent placement can be a safe alternative tool.

## Introduction

As early gastric cancer (EGC) is known to have a low recurrence rate and a long survival time after treatment [1], greater emphasis has been placed on developing function-preserving and less invasive treatment methods to improve the post-treatment quality of life (QOL). Pylorus-preserving gastrectomy (PPG) has recently been recognized as a function-preserving surgery for patients with EGCs located in the middle third of the stomach as it can provide nutritional and immunological benefits while providing a similar level of surgical and oncological safety as conventional distal gastrectomy (DG) [2–7]. Indeed, many previous studies have reported several functional and nutritional advantages of PPG over conventional DG such as the lower frequency of the dumping syndrome, gastritis, bile reflux, gallstone formation, and impaired weight gain, resulting in a better QOL [3–7].

Preservation of pyloric function is an essential concept of PPG, in which meticulous caution is paid to maintain intact innervation and blood supply to the pylorus [8]. However, preservation of these nerves and vessels can lead to limitations or even omissions of lymph node dissections which may compromise its radicality, eventually raising oncologic concerns. To overcome this oncologic problem, excessive dissection is often performed during PPG, at a cost of injury to the innervation or blood supply to the pylorus. Therefore, it has been reported that owing to the resultant impaired pyloric function, patients may occasionally experience a sensation of gastric fullness after food intake as well as long-term retention of food in the remnant stomach [9–11]. Indeed, this delayed gastric emptying caused by pyloric spasms has been shown to decrease the patient’s QOL and has also been shown to interfere with the detection of secondary cancers in the gastric remnant on endoscopy or double contrast barium studies [12].

Although these pyloric spasms following PPG have been increasingly recognized, there is no standardized management strategy at present, and thus gastric surgeons have often found themselves hesitant to perform this minimally invasive, function-preserving surgery. Until now, fluoroscopy-guided balloon dilatation or retrievable stent insertion has been increasingly used to alleviate benign gastrointestinal tract obstructions [13–18], yet there have been no reports describing the feasibility or clinical usefulness of these interventional procedures for pyloric spasms after PPG.

Therefore, the purpose of this study was to retrospectively investigate the feasibility and clinical usefulness of fluoroscopy-guided balloon dilatation or retrievable stent insertion for pyloric spasms after PPG.

## Materials and Methods

This retrospective study was approved by Seoul National University Hospital Institutional Review Board under approval number H-1411-024-623 and the requirement for informed consent was waived. All patients’ characteristics and results of subjective and objective analyses were anonymized and de-identified prior to statistical analysis.

### Patients

**Fig 1:**
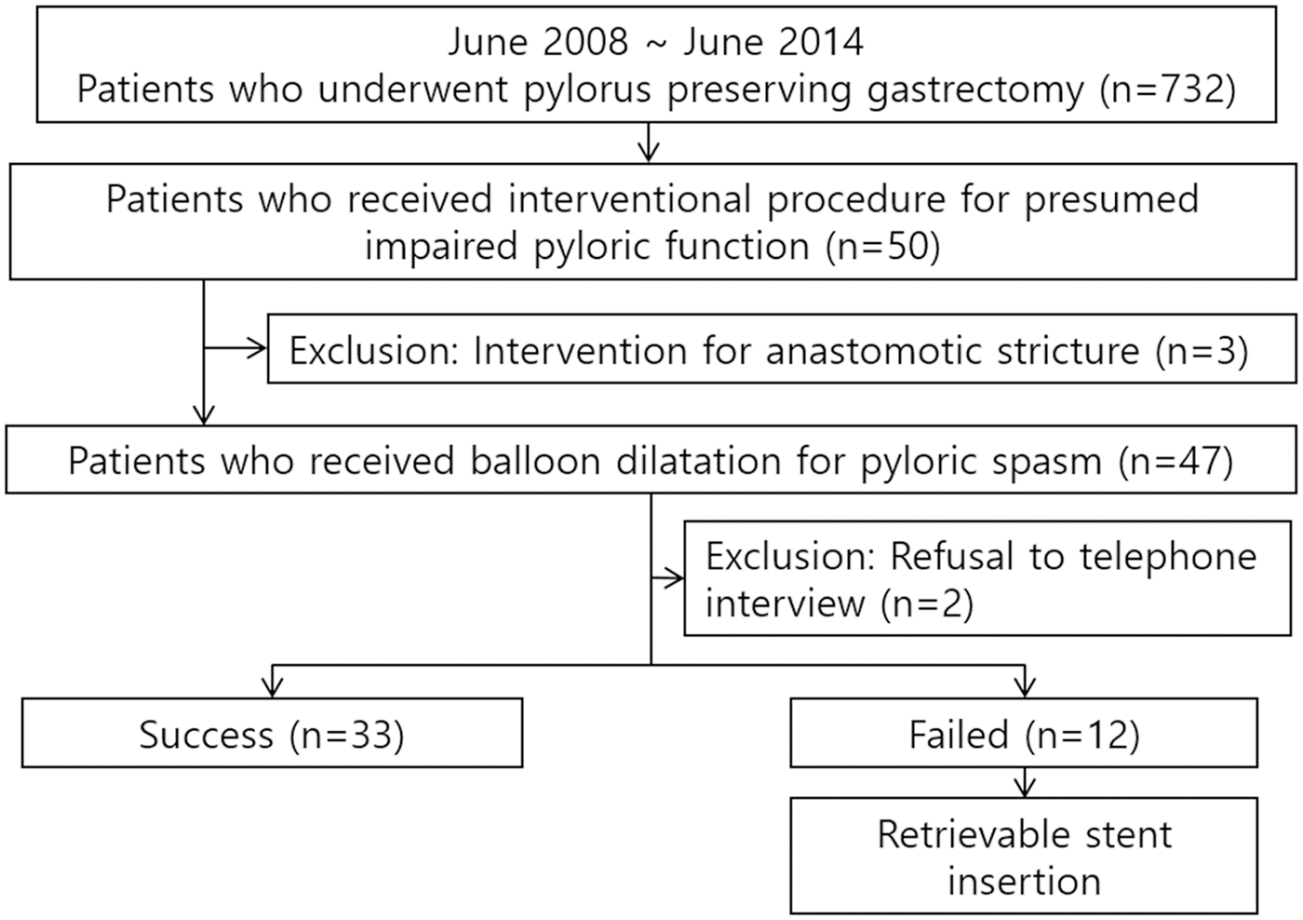
A flow diagram of our study population.

From our hospital’s electronic medical records and radiology database between June 2008 and June 2014, we collected a list of 732 patients who underwent PPG. Among them, 50 patients underwent balloon dilatation and/or retrievable stent insertion for presumed impaired pyloric function after PPG. Of them, 3 patients were excluded as they had received interventional treatment for anastomotic strictures. Thus, finally, 47 patients (23 males, 24 females; mean age, 60.7 years; range, 37–86 years) comprised our study population (S1 File). A detailed summary of the patients’ characteristics can be found in Table 1. Fig 1 shows a flow diagram of our patients.

**Table 1:** Patients’ demographics for the initial 47 and final 45 patients.

Characteristics		Values for initial 47 patients	Values for final 45 patients
M:F		23:24	22:23
Mean age (age range)		60.7 ± 11.2 years (37~86 years)	60.9 ± 11.4 years (37~86 years)
Type of operation	Open	3	3
	Laparoscopy-assisted	41	40
	Robot-assisted	3	2
Pathologic T staging	T1a	34	33
	T1b	10	10
	T2	2	1
	T3	1	1
Mean interval between surgery and procedure	Balloon dilatation	28.7 ± 35.7 days (8~171 days)	25.4 ± 31.6 days (8~171 days)
	Retrievable stent insertion	20.3 ± 6.0 days (10~33 days)	20.7 ± 6.2 days (10~33 days)

Numbers in parentheses are ranges

**Fig 2:**
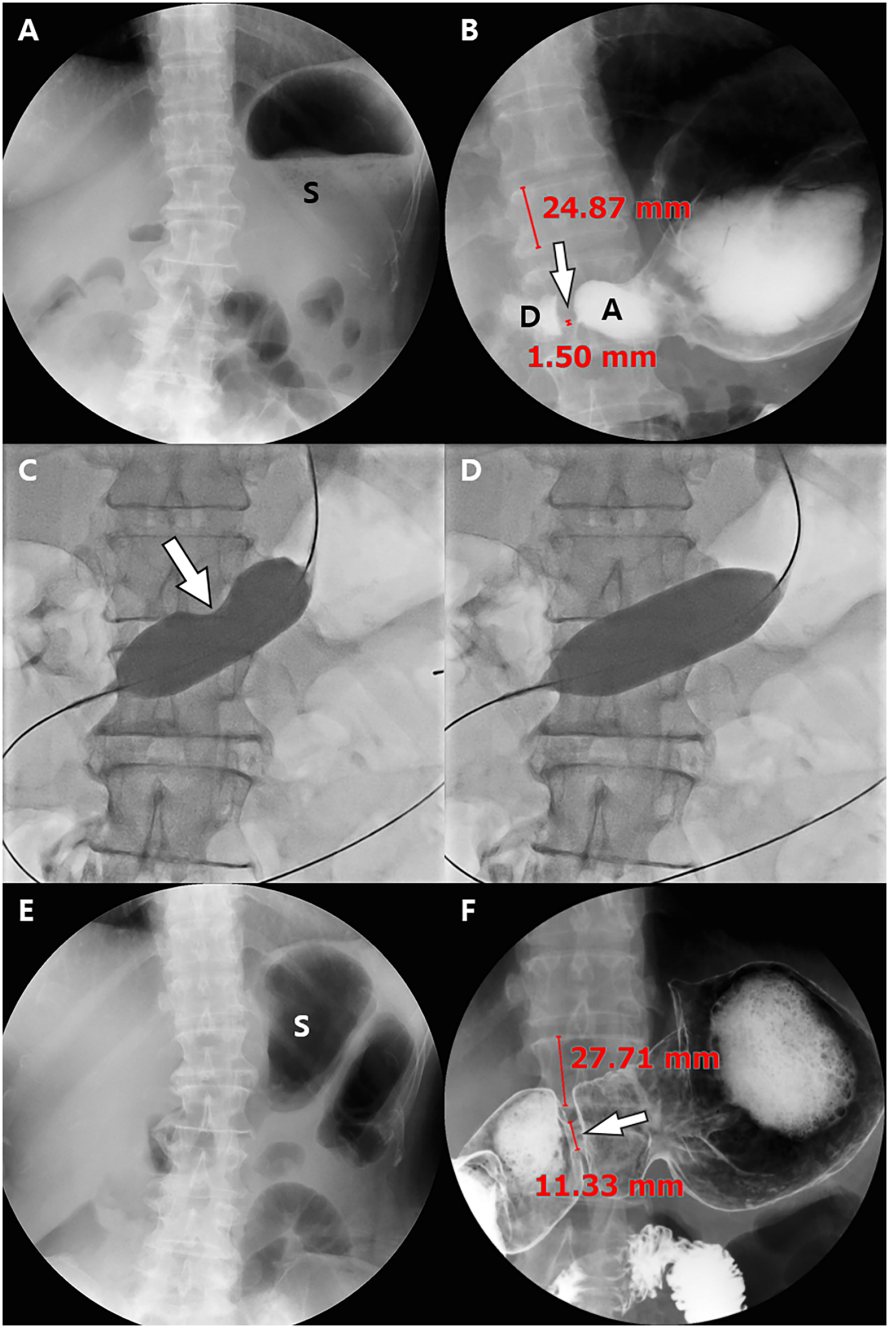
Images of a 60-year-old woman with impaired pyloric function after laparoscopy-assisted pylorus-preserving gastrectomy (PPG). (A) On the scout image obtained 13 days after PPG, approximately 1/2~3/4 of the stomach (S) is filled with residual food material, resulting in a semi-quantitative score for residual food of 2. (B) On upper gastrointestinal series (UGIS), the pyloric canal is severely narrowed (arrow) and the width of the pyloric canal and the height of the adjacent vertebral body are 1.5 and 24.87, respectively, with a pyloric canal-to-height of vertebral body ratio of 6.03. A = antrum, D = duodenum. (C), (D) Fluoroscopic images show dilatation of the stenotic pyloric canal with a 25 mm x 4 cm long balloon until the balloon deformity disappears. (E) The scout image of UGIS obtained 11 months after balloon dilatation shows no residual food material within the stomach, resulting in a markedly improved semi-quantitative score for residual food of 5. (F) UGIS shows a dilated pyloric canal (arrow), and the width of the pyloric canal and the height of the adjacent vertebral body are 11.33 and 27.71, respectively. Therefore, the pyloric canal-to-height of vertebral body ratio was also markedly increased to 40.89. Finally, her subjective symptom score of post-prandial discomfort was also markedly decreased from 10 to 3 after the balloon procedure.

**Fig 3:**
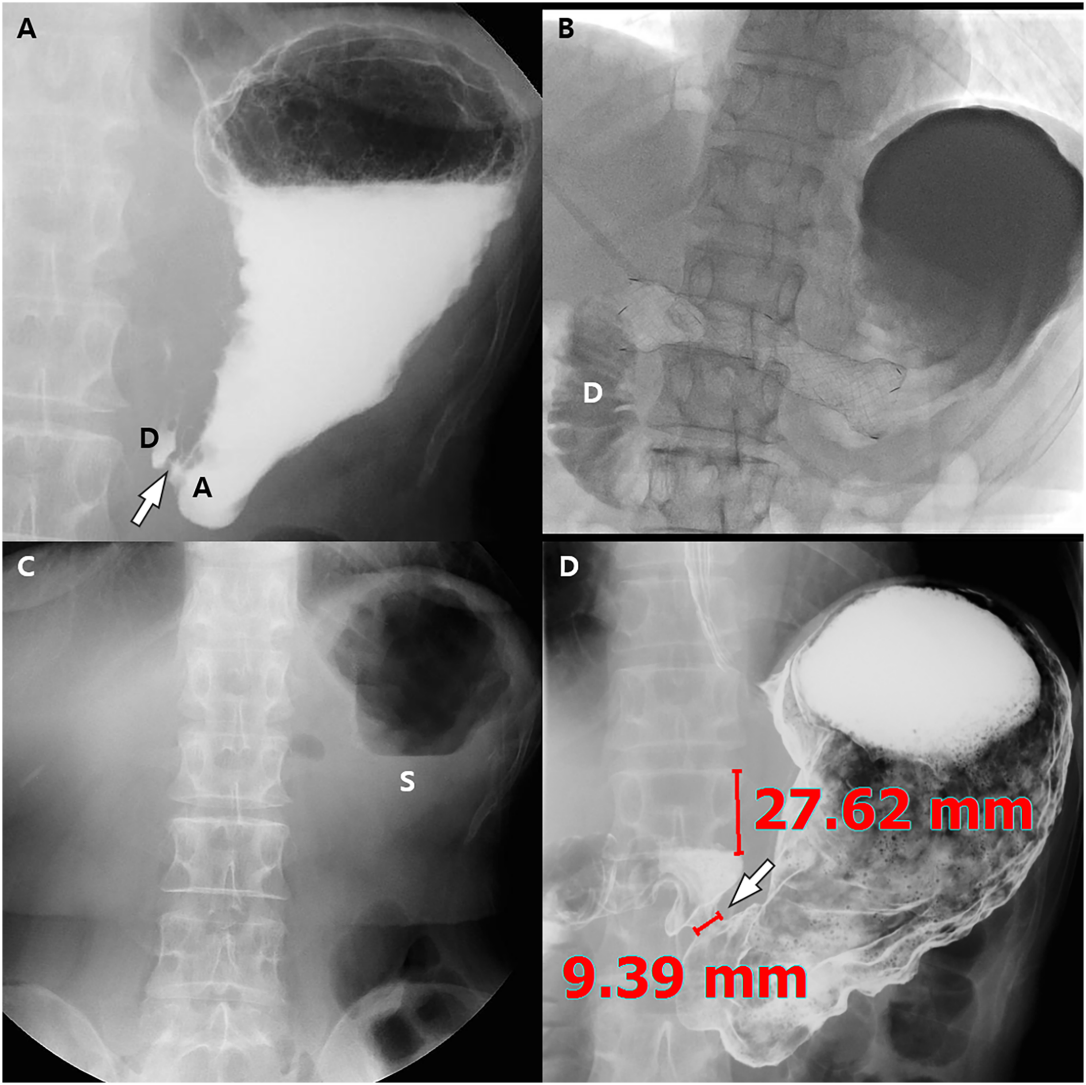
Images of a 68-year-old woman with impaired pyloric function after laparoscopy-assisted pylorus-preserving gastrectomy (PPG). She underwent balloon dilatation with a 22 mm x 4 cm long balloon 3 times for 3 minutes with a 1-minute interval 19 days after PPG. (A) Post-ballooning upper gastrointestinal series (UGIS) shows tight stenosis of the pyloric canal (arrow) due to recoil of the stenosis. The pyloric canal-to-height of vertebral body ratio was 3.7. (B) Stent insertion was done for pyloric spasms with a 20 mm x 10 cm long fully covered retrievable stent. Fluoroscopic image shows good passage of the contrast agent to the duodenum (D). Stent removal was successfully done 2 weeks after stent insertion. (C) On the scout UGIS obtained 3 months after stent removal, approximately 1/4~1/2 of the stomach (S) is filled with residual food material, resulting in a semi-quantitative score for residual food of 3. (D) UGIS shows a dilated pyloric canal (arrow) and the width of the pyloric canal and the height of the adjacent vertebral body are 9.39 and 27.62, respectively. Therefore, the pyloric canal-to-height of vertebral body ratio was markedly increased from 3.7 to 34.0. Finally, her subjective symptom score for post-prandial discomfort was also markedly resolved from 10 to 3 after the stent procedure.

Impaired pyloric function was diagnosed using the typical imaging feature of short segmental and persistent narrowing of the pylorus on upper gastrointestinal series (UGIS) (Figs 2 and 3) as well as by the presence of symptoms such as post-prandial epigastric fullness and indigestion due to delayed gastric emptying. In all patients, balloon dilatation was initially performed. The patients were then divided into two groups; the balloon group and the stent group. Patients who showed good response to balloon dilatation requiring no further treatment until the end of follow-up were classified into the balloon group whereas patients who showed no or poor response to balloon dilation requiring subsequent retrievable stent placements were classified into the stent group.

### Gastric Balloon Dilatation Technique

The balloon dilation technique performed in our hospital was similar to that reported in previous studies [16, 19]. In brief, a 0.035-inch hydrophilic guide wire was inserted under fluoroscopic guidance, across the stenotic pyloric canal, and into the duodenum or jejunum. Thereafter, a 4~6 cm-long, 16~25 mm-diameter noncompliant, low-pressure balloon catheter was passed over the guide wire to a position over the stenotic pyloric canal. The balloon was then slowly inflated using hand pressure or an insufflator (with 2~8 atms) for 1 minute with a diluted water-soluble contrast medium until the “hourglass deformity” created by the stenotic pyloric canal disappeared from the balloon contour or until the patient could not tolerate further inflation (Fig 2). The inflation was repeated 1 to 12 times with 1-minute intervals between inflations in one session. After the procedure, the presence of immediate complications such as an extraluminal leakage of contrast material was checked.

### Retrievable Stent Placement and Removal Technique

The stent placement techniques used in our hospital have been described previously [19, 20]. In brief, a stiff angled, 260 cm long, 0.035-inch stiff guide wire was inserted under fluoroscopic guidance across the stenotic pyloric canal and into the duodenum or jejunum. The stent delivery system was then advanced over the guide wire, passing it through the obstruction, and the 8~15 cm long, 20 mm-diameter fully covered retrievable stent (Niti-S, Taewoong Medical, Seoul, South Korea) was released over the obstruction (Fig 3). If the stent was not well inflated, balloon insufflation with a 20 mm x 4 cm balloon was performed within the stent. In this stent delivery system, one end of a string is attached to the proximal edge of the stent and the other end is passed through the stomach and esophagus, exiting through nostril, and is finally anchored around the ear. This string is used to prevent distal migration of the stent as well as to facilitate stent removal.

**Fig 4:**
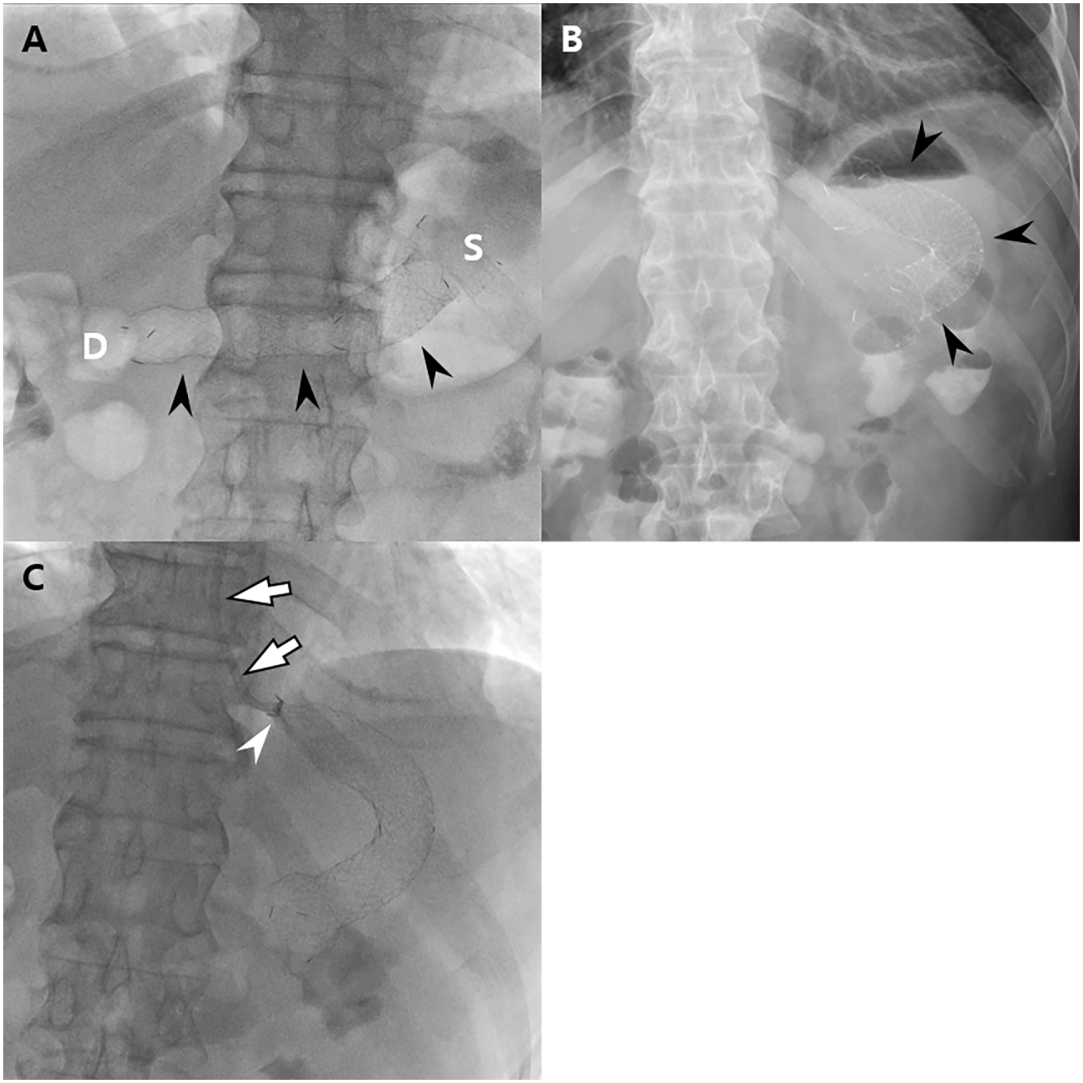
Images of a 56-year-old man with impaired pyloric function after laparoscopy-assisted pylorus-preserving gastrectomy (PPG). The patient underwent balloon dilatation and subsequent stent insertion 21 and 23 days after PPG, respectively. (A) On the scout image obtained after stent insertion, the stent (arrowheads) is well placed extending from the stomach (S) to the duodenum (D) with its center located at the pyloric canal. (B) On plain abdominal radiograph obtained after 1 day, the stent (arrowheads) has migrated proximally and is located within the remnant stomach. (C) Fluoroscopic guided stent removal was done using an angiographic catheter (arrows). Note the collapsed proximal end (arrowhead) of the stent.

Removal of the fully covered retrievable stent was performed 1~2 weeks after temporary stent placement or in cases of stent-related complications such as stent migration under fluoroscopic guidance exploiting the previously inserted string (Fig 4). After the procedure of stent insertion and removal, the presence of immediate complications such as an extraluminal leakage of contrast material was also checked.

### Data Analysis

Telephone interviews were conducted to evaluate the clinical success of the procedure on October 2014. Every patient received our phone calls just once. The patients were asked questions regarding post-prandial symptoms such as epigastric fullness and indigestion on a 10-point scale (1: no symptom, 10: extreme discomfort) before and after the intervention. We modified a 5-point Likert dyspepsia scale (1, no complaints; 2, few complaints; 3, moderate complaints; 4, many complaints; and 5, serious complaints that significantly affect daily life) to a 10-point scale [21]. Clinical success was determined as a decrease in score of ≥ 3 points after the procedure, without additional treatment until the last follow-up. All patients except two responded to the telephone interview; one patient died of co-morbid cardiac disease 1 year after PPG and balloon dilatation and the other patient refused to participate in the interview. Therefore, those two patients were excluded from further analysis of our data.

**Fig 5:**
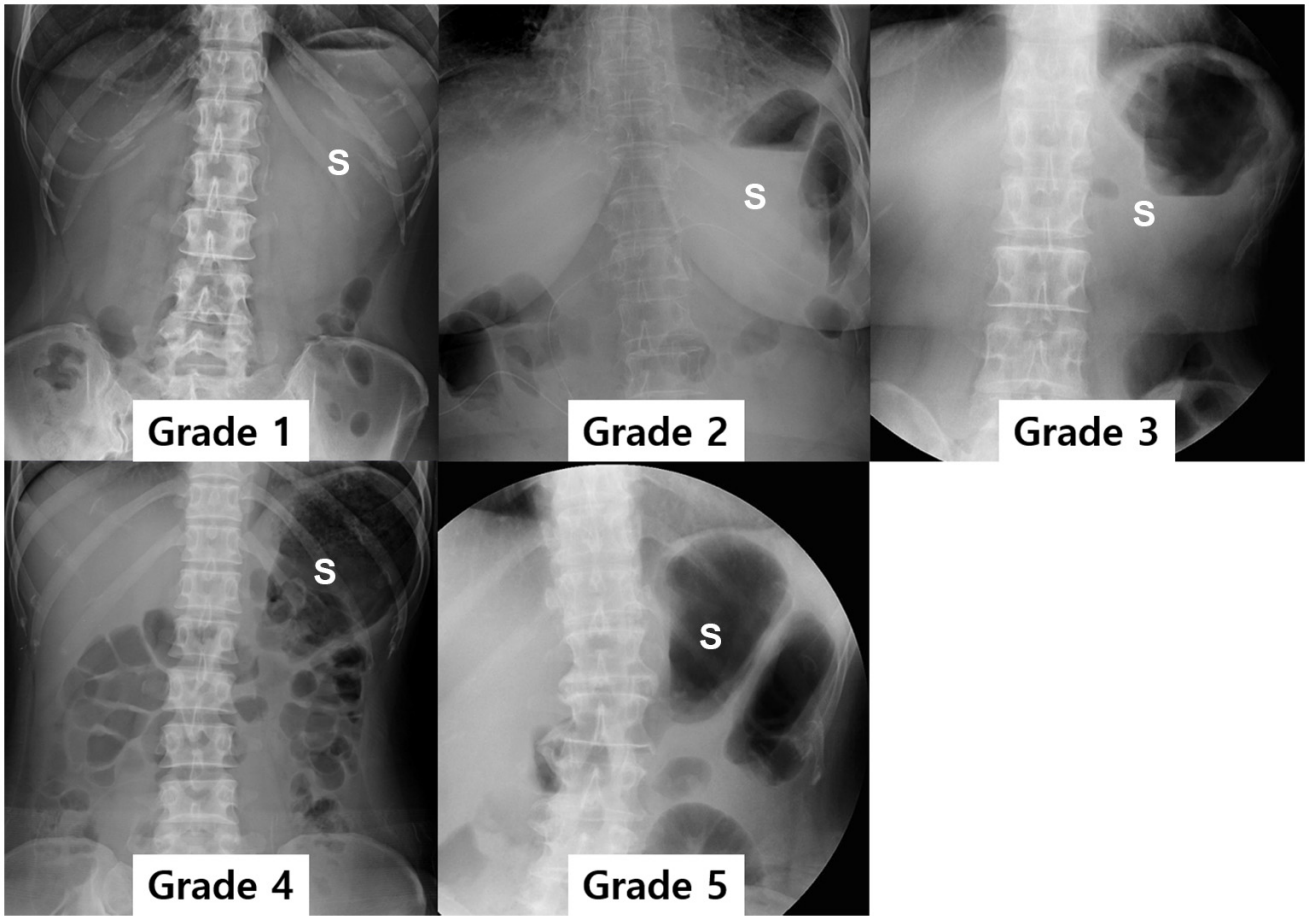
Semi-quantitative grading for the degree of residual food stagnation within the remnant stomach. Grade 1 is defined as when more than 3/4 of the remnant stomach is filled with residual food; grade 2 when 3/4 ~ 1/2 of the residual stomach is filled; grade 3 when 1/2 ~ 1/4 of the residual stomach is filled; grade 4 when less than 1/4 of the residual stomach is filled; grade 5 when the entire stomach is empty on the scout image of the upper gastrointestinal series.

For quantitative and semi-quantitative evaluation of treatment response, two radiologists (JSB with two years of experience and SHK with 17 years of experience) reviewed the UGIS or pneumo-CT before and after interventional treatment to measure the diameter of the pylorus as well as the height of the adjacent vertebral body so as to calculate the ratio of the pyloric canal-to-the height of the vertebral body (Figs 2 and 3), in a consensus manner. In addition, they graded the degree of residual food stagnation on a 5 point scale (1, >3/4 opacification; 2, ≤3/4—>1/2 opacification; 3, ≤1/2—>1/4 opacification; 4, ≤1/4 opacification; 5, empty) on a scout image of UGIS or CT (Fig 5).

### Statistical Analysis

All patients’ characteristics and results of subjective and objective analyses were anonymized and de-identified prior to statistical analysis. Then we compared these variables between the balloon and stent groups using univariate statistical analyses: chi-square or Fisher’s exact test for categorical variables and the Student t or Mann-Whitney U test for continuous variables. In each group, subjective symptom scores as well as quantitative and semi-quantitative data were also compared before and after interventional treatments using the paired t-test or Wilcoxon signed rank test. A two-sided P value of less than 0.05 was considered to indicate statistical significance. All statistical analyses were performed using a commercially available software package (SPSS version 21; SPSS, Chicago, Ill).

## Results

The characteristics of final 45 patients are summarized in Table 1. All interventional treatments of the 45 included patients were clinically successful; the mean subjective symptom score of these 45 patients was significantly improved from 9.04 ± 2.06 prior to treatment to 1.93 ± 1.56 after treatment (P <.0001). The mean follow-up period of these 45 patients was 26.2 months (range, 4–75 months) and no patients showed recurrence of symptoms related to pyloric spasms.

In addition, of the 45 patients, 33 (73.3%, 33/45) showed good response after one (n = 29) or two (n = 4) sessions of balloon dilatations and required no further treatment until the end of follow-up (balloon group). Conversely, 12 patients (26.7%, 12/45) had a poor response after balloon dilatation and required retrievable stent insertion (stent group). Fig 1 shows a flow diagram of our patients. At univariate analysis, pre-ballooning subjective scores for epigastric fullness was significantly worse in the stent group (9.8 ± 0.6) than in the balloon group (8.8 ± 2.3) (P = .018) (Table 2). Other parameters were not significantly different between the two groups (Table 2). Table 3 represents details regarding follow-up period in both groups.

**Table 2:** Results of Univariate Statistical Analysis between Balloon and Stent Groups.

	Parameters	Balloon group (n = 33)	Stent group (n = 12)	P value
	M:F	15:18	7:5	0.445
	Mean Age ± SD (years)	60.4 ± 12.0	62.3 ± 9.9	0.625
Type of operation	Open: Laparoscopy-assisted: Robot-assisted	3:29:1	0:11:1	0.436
Pathologic T stage	T1a:T1b:≥T2	24:7:2	9:3:0	0.674
	Mean interval between surgery and balloon dilatation	29.1 ± 36.2	15.4 ± 5.7	0.202
	Balloon diameter	20.7 ± 2.4	20.3 ± 0.8	0.411
	Number of balloon sessions	1.1 ± 0.3	1.1 ± 0.3	0.728
Pre-ballooning	Subjective symptom score	8.8 ± 2.3	9.8 ± 0.6	0.018
	Ratio of pyloric canal-to-height of vertebral body	7.2 ± 6.1	5.8 ± 6.4	0.495
	Semi-quantitative score for food retention	1.8 ± 0.7	1.7 ± 0.5	0.395

Ratio of pyloric canal-to-height of vertebral body = (diameter of pyloric canal) / (height of adjacent vertebral body) × 100

**Table 3:** Number of Patients according to Follow-up Period in Each Group.

	Balloon Group (n = 33)	Stent Group (n = 12)
< 6 months	1	2
6–12 months	5	4
13–18 months	4	1
19–24 months	6	3
25–36 months	7	1
> 36 months	10	1

### Balloon Group (n = 33)

In the 33 patients who showed good response to balloon dilatations and did not require additional treatment, a total of 37 balloon dilatation sessions were successfully performed: a single session in 29 patients and two balloon sessions in the remaining 4 patients (Fig 2). The mean interval between surgery and balloon dilatations was 29.1 ± 36.2 days (range, 8–171 days). All balloon dilatations were successfully performed without immediate complications.

**Fig 6:**
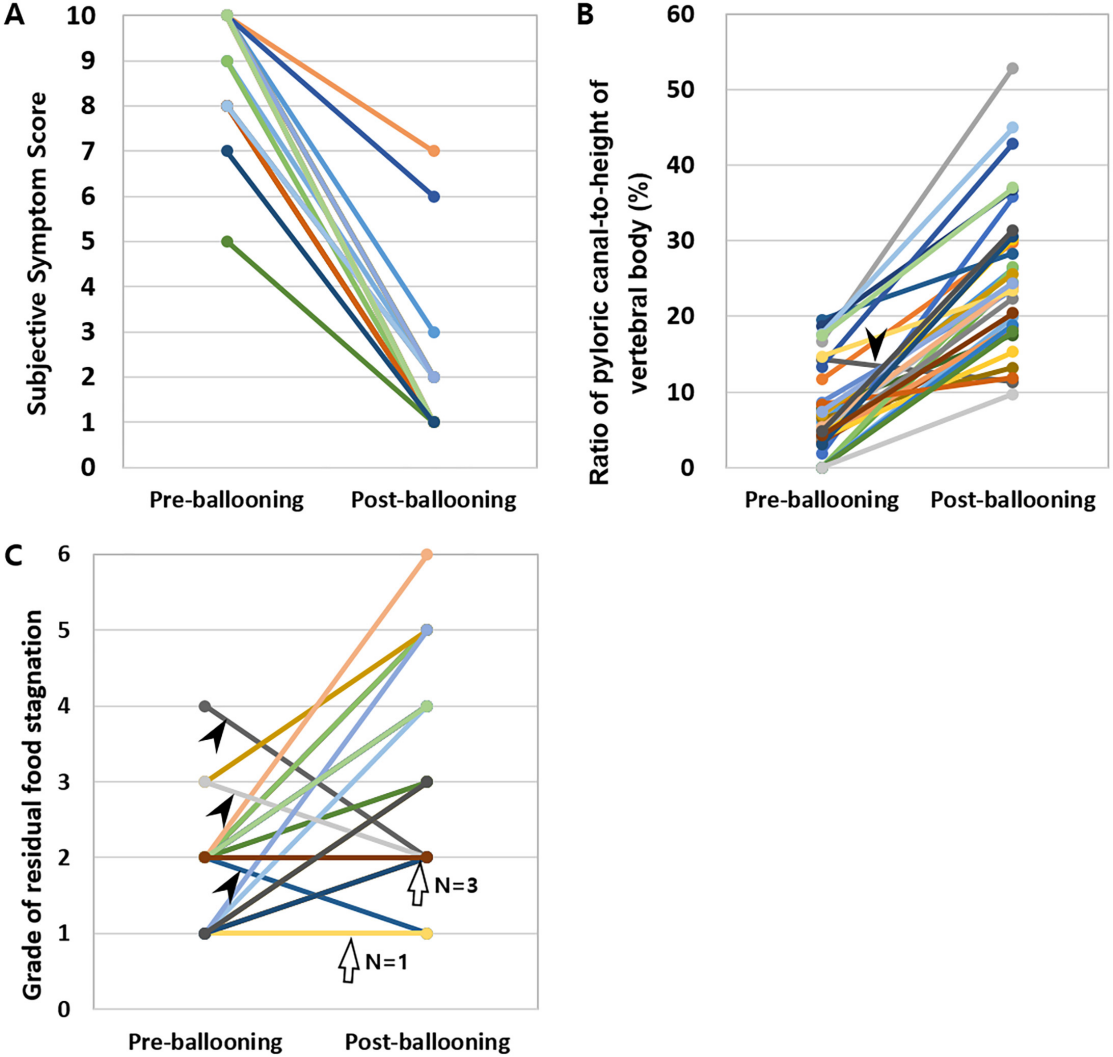
Graphs showing the results of subjective and objective analyses in the balloon group. (A) Plotting of subjective symptom scores before and after balloon dilatation. In all patients, subjective symptom scores improved after balloon dilatation. (B) Plotting of the pyloric canal-to-height of vertebral body ratio before and after balloon dilatation. In all patients except one (arrow), the pyloric canal-to-height of vertebral body ratio increased after balloon dilatation. (C) Plotting of the grade for residual food stagnation before and after balloon dilatation. In 26 of 33 patients, the grade of residual food stagnation improved. The grade of residual food stagnation was the same after balloon dilatation in four patients (arrows) (2 to 2 in three and 1 to 1 in one) and became worse in the remaining three (arrowheads) (2 to 1, 3 to 2, and 4 to 2).

Compared to pre-ballooning, the mean score of subjective discomfort was significantly improved after balloon dilatation (8.8 ± 2.3 versus 1.7 ± 1.4) (P <.0001). In all patients except two, subjective symptom scores after balloon dilatation decreased to 5 or less (Fig 6). In all patients except one, the ratio of the pyloric canal-to-height of vertebral body increased after balloon dilatation (Fig 6). The mean ratio of the pyloric canal-to-height of vertebral body was significantly increased after the balloon procedures (7.2 ± 6.1 versus 25.4 ± 9.9) (P <.0001). In addition, the mean grade for residual food stagnation within the remnant stomach was also improved from 1.8 ± 0.7 prior to balloon dilatation to 2.5 ± 1.3 after ballooning (P <.0001). In most patients (26/33, 78.8%), the grade of residual food stagnation improved by at least 1 (Fig 6).

### Stent Group (n = 12)

In all 12 patients, stent placement was successfully performed without immediate complications. Of the 12 patients (26.7%) who showed poor or no response after one (n = 11) or two (n = 1) sessions of balloon dilatation necessitating retrievable stent insertions (Fig 3), all balloon procedures except one were technically successful without immediate complications. In one patient who underwent laparoscopy-assisted PPG and subsequent balloon dilatation using a 20 mm x 4 cm balloon 4 days after surgery, a small transmural tear (appearing as extraluminal leakage of contrast media at follow-up UGIS) occurred around the anastomotic site. The transmural tear resolved within 5 days after conservative management including fasting and antibiotics. However, her symptom of epigastric fullness was not improved and therefore, subsequent stent insertion was finally performed.

The mean interval between surgery and initial balloon dilatations was 15.4 ± 5.7 days (range, 4–21 days) and the mean interval between surgery and stent insertions was 20.7 ± 6.2 days (range, 10–33 days).

In 3 of 12 patients (30.77%), stent migration occurred 1, 3, and 8 days after stent placement (Fig 4). Two stents migrated proximally and one migrated distally. All three stents were successfully removed under fluoroscopic (n = 2) and endoscopic (n = 1) guidance without any complications. The stent was then re-inserted in 2 of these 3 patients owing to the persistence of symptoms, whereas in the remaining 1 patient, stent reinsertion was not done because of an improvement of the patient’s clinical symptoms. The mean duration of the stent retention time was 10.4 ± 5.0 days (range, 3–18 days). Finally, all stents were successfully removed.

**Fig 7:**
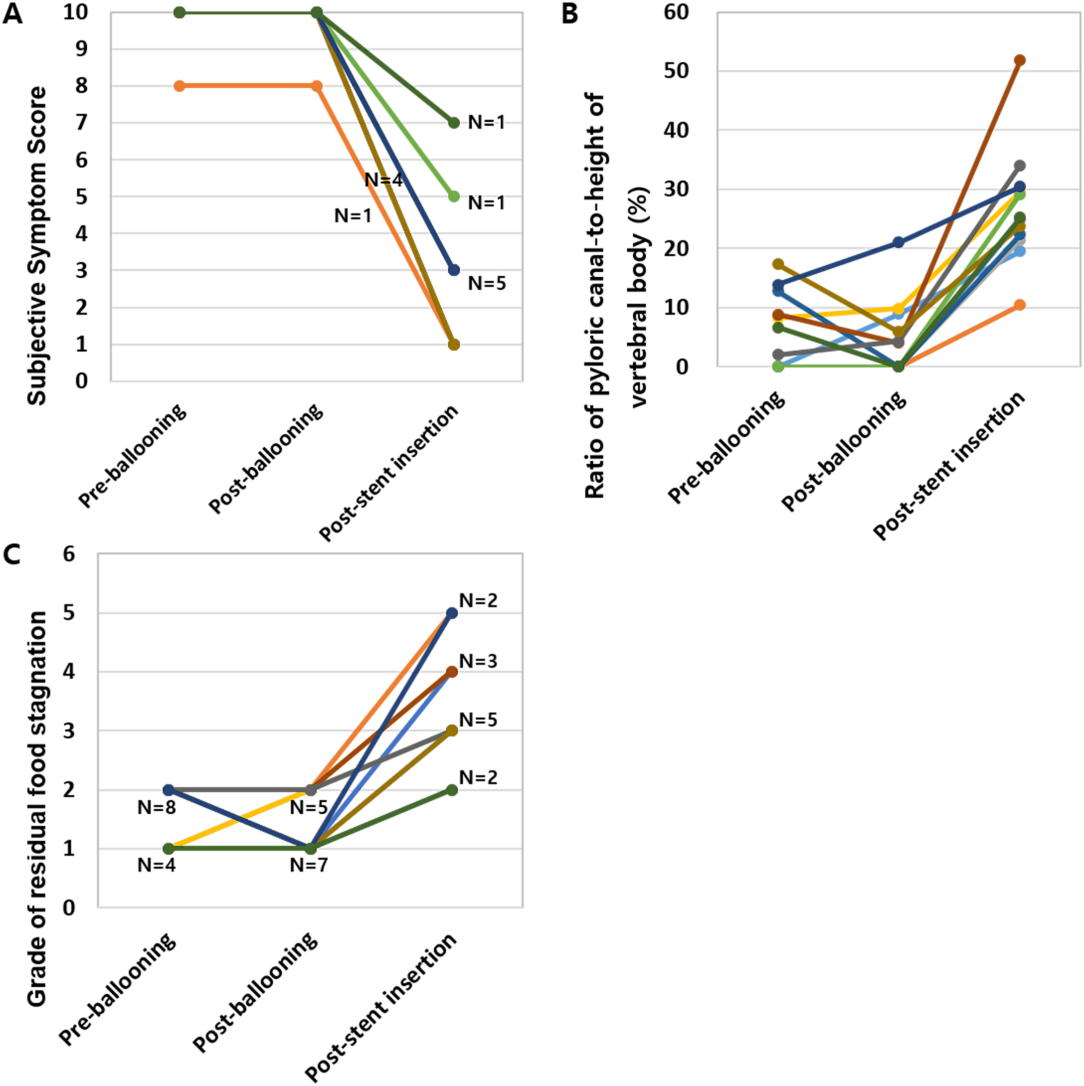
Graphs showing the results of subjective and objective analyses in the stent group. (A) Plotting of subjective symptom scores before and after balloon dilatation and after stent insertion. In all patients, subjective symptom scores were not improved after balloon dilatation. However, it was significantly improved after stent insertion. (B) Plotting of the pyloric canal-to-height of vertebral body ratio before and after balloon dilatation and after stent insertion. In all patients, the ratio of the pyloric canal-to-height of the vertebral body increased after stent insertion compared to both before and after balloon dilatation. (C) Plotting of the grade for residual food stagnation before and after balloon dilatation and after stent insertion. In all patients, the grade of residual food stagnation improved after stent insertion compared to both before and after balloon dilatation.

In all 12 patients, the mean subjective symptom score did not improve after balloon dilatation (9.8 ± 5.8 versus 9.8 ± 5.8) (P = 1.000). However, after stent insertion, the mean symptom score significantly improved to 2.7 ± 1.9 (P = .002) (Fig 7). In addition, although the mean ratio of the pyloric canal-to-height of the vertebral body was not significantly different before and after the balloon procedure (5.8 ± 6.4 versus 4.5 ± 6.4) (P = .575), after stent insertion, the mean ratio significantly increased to 26.9 ± 1.0 (P = .002) (Fig 7). A similar trend was observed for the grade of residual food stagnation (Fig 7).

## Discussion

Despite of the many clinical and nutritional benefits of PPG over conventional DG, it has been well-known that PPG can cause a feeling of epigastric fullness after meals along with delayed gastric emptying owing to impaired pyloric function. Indeed, our retrospective study results demonstrated that pyloric spasms were able to be observed in the early post-operative period in 6.4% (47/732) of patients who underwent PPG, which is well in line with the results of previous studies in which significant or continuous gastric stasis symptoms were reported in 6.2% to 23% of patients who had undergone PPG [7, 10, 22–25]. As this resultant sensation of epigastric fullness induced by impaired pyloric function may lead to a significant decrease in the QOL as well as lead to difficulty in detecting secondary cancers [26], appropriate and early treatment of this condition would provide a huge benefit to patients who suffer from this condition after PPG.

In our study, fluoroscopy-guided balloon dilatation was able to resolve the obstructive symptoms of 73.3% (33/45) of our patients. In the remaining patients who did not respond to balloon dilatation, retrievable stents were able to improve their obstructive symptoms. The mean subjective symptom score improved significantly from 9.0 to 1.9 after both procedures. The treatment effect was also quantitatively and semi-quantitatively proven by measuring the ratio of the pyloric canal-to-the height of the adjacent vertebral body and by grading the degree of residual food stagnation, respectively. Furthermore, no patients showed recurrence of pyloric spasms during a mean follow up of 26.2 months (range, 4–75 months). To the best of our knowledge, this is the first report showing the long-term results of fluoroscopy-guided balloon dilatation or retrievable stent insertion for the purposes of alleviating pyloric spasms after PPG.

Our study results showed that balloon dilatation can be a safe and effective first-line treatment option for pyloric spasms after PPG owing to its high success and low complication rate. In terms of complications, only one transmural tear was observed among the 50 balloon dilatation sessions (2%, 1/50) performed in both balloon and stent groups and even it spontaneously resolved through only conservative treatment. Considering the high success rate and low complication rate of balloon dilatations as shown in our study, balloon dilatations should be chosen as a first-line treatment option for pyloric spasms after PPG.

Although most patients responded to the balloon dilatation, more than 20% of our patients did not show improvement of their obstructive symptoms, owing to recoiling of the pyloric canal. Our study results showed that implementing retrievable stent insertion was helpful in resolving these symptoms in patients refractory to balloon dilatations. This result may be of huge clinical significance as finding another effective and non-invasive treatment option in patients with pyloric spasms refractory to initial balloon dilatations remains an important therapeutic challenge.

The theoretical benefit of temporary retrievable stenting may be explained by its extended dilation process in which a gradually-enhancing force is exerted to the wall of the gastrointestinal tract for up to several days or weeks [14]. Considering that the dilation strength of stenting would be better distributed and persistent than with balloon dilation [17], stents may lead to a good clinical outcome in patients who failed to achieve resolution of pyloric spasms due to recoiling of the pyloric canal. However, a major drawback of stent placement in a benign narrowing may be stent migration. In general, benign strictures are short in length and somewhat smooth in contour, increasing the likelihood of stent migration [27]. Furthermore, a fully covered retrievable stent is insufficiently anchored to the intestinal wall [28], thus increasing the possibility of stent migration. In our study, retrievable stents were migrated in three patients (25%, 3/12) prior to their scheduled elective stent removal. Two stents were migrated proximally and one was migrated distally although a long thread connected to the proximal part of the stent which was anchored to the patients’ ear to prevent downward migration of the stent. Even though migrated stents were successfully able to be removed using a fluoroscopic device or an endoscope without any complications, the development of newer stents such as dual or conformable stents is strongly warranted to minimize stent migration. Considering the relatively more invasive nature and a higher migration rate of stent insertion over balloon dilatations, retrievable stent insertions should be considered if the initial balloon dilatation is not successful.

Another important factor affecting the clinical outcome and patient compliance related to stent insertion is the stent retention time. Previous studies have reported that the mean stent placement periods varied between 4 days—8 weeks if no complications were evident [14, 29–31]. In our study, the mean stent retention time was 10.4 days after stent insertion. Therefore, we believe that continuous dilation with a stent for approximately 1.5 weeks may be sufficient to adequately tear the pyloric canal resulting in a relatively good treatment outcome while avoiding difficulties in retrieval.

Our study has several limitations. First, because our study was retrospectively designed, the study protocols were not well standardized and important information such as the balloon pressure or ballooning time was missing from the medical and radiologic reports. In addition, we measured the diameter of the pyloric canal using gastrografin in a single contrast study during the pre-procedural period in all of our patients, while it was measured using a double contrast barium study (n = 25) or pneumo-CT (n = 9) during the post-procedural period in 45 patients. Thus, the use of an effervescent agent as a double contrast agent may have overestimated the diameter of the pyloric canal. Therefore, further prospectively designed studies are warranted to provide a stronger conclusion. Second, although we attempted to perform a telephone interview to overcome the limitations of radiologic evaluation, the information provided by the patients might have been subject to recall-bias. Such study limitations may limit the validity of our study results. Third, as we recruited only patients who received interventional treatment, natural history of pyloric spasm after PPG has not been investigated. For example, we were not able to answer the question what proportion of these patients would be expected to improve with conservative medical treatment. Further prospective study regarding this specific issue should be conducted in the future.

In conclusion, fluoroscopy-guided balloon dilation is a safe and effective first-line treatment option for patients with pyloric spasms after PPG. In addition, for patients refractory to balloon dilations, retrievable stent placement can be a safe alternative tool.

## Supporting Information

S1 File Data of patients of the balloon group and the stent group. (XLS)

## Abbreviations

PPG: pylorus-preserving gastrectomy
DG: distal gastrectomy
EGC: early gastric cancer
QOL: quality of life
UGIS: upper gastrointestinal series
